# Randomized comparison of next-generation sequencing and array comparative genomic hybridization for preimplantation genetic screening: a pilot study

**DOI:** 10.1186/s12920-015-0110-4

**Published:** 2015-06-23

**Authors:** Zhihong Yang, James Lin, John Zhang, Wai Ieng Fong, Pei Li, Rong Zhao, Xiaohong Liu, William Podevin, Yanping Kuang, Jiaen Liu

**Affiliations:** ZytoGen Global Genetics Institute, Timonium, MD USA; Reproductive Fertility Center, Irvine, CA USA; New Hope Fertility Center, New York, NY USA; Hospital Conde S. Januário, Macau, P. R. China; Jia En De Yun Hospital, Beijing, P. R. China; Pacific Reproductive Center, Torrance, CA USA; Ninth People’s Hospital, Shanghai Jiao Tong University School of Medicine, Shanghai, P. R. China

**Keywords:** NGS, aCGH, PGS, Aneuploidy screening, Ongoing pregnancy, Implantation

## Abstract

**Background:**

Recent advances in next-generation sequencing (NGS) have provided new methods for preimplantation genetic screening (PGS) of human embryos from in vitro fertilization (IVF) cycles. However, there is still limited information about clinical applications of NGS in IVF and PGS (IVF-PGS) treatments. The present study aimed to investigate the effects of NGS screening on clinical pregnancy and implantation outcomes for PGS patients in comparison to array comparative genomic hybridization (aCGH) screening.

**Methods:**

This study was performed in two phases. Phase I study evaluated the accuracy of NGS for aneuploidy screening in comparison to aCGH. Whole-genome amplification (WGA) products (n = 164) derived from previous IVF-PGS cycles (n = 38) were retrospectively analyzed with NGS. The NGS results were then compared with those of aCGH. Phase II study further compared clinical pregnancy and implantation outcomes between NGS and aCGH for IVF-PGS patients. A total of 172 patients at mean age 35.2 ± 3.5 years were randomized into two groups: 1) NGS (Group A): patients (n = 86) had embryos screened with NGS and 2) aCGH (Group B): patients (n = 86) had embryos screened with aCGH. For both groups, blastocysts were vitrified after trophectoderm biopsy. One to two euploid blastocysts were thawed and transferred to individual patients primarily based on the PGS results. Ongoing pregnancy and implantation rates were compared between the two study groups.

**Results:**

NGS detected all types of aneuploidies of human blastocysts accurately and provided a 100 % 24-chromosome diagnosis consistency with the highly validated aCGH method. Moreover, NGS screening identified euploid blastocysts for transfer and resulted in similarly high ongoing pregnancy rates for PGS patients compared to aCGH screening (74.7 % vs. 69.2 %, respectively, *p* >0.05). The observed implantation rates were also comparable between the NGS and aCGH groups (70.5 % vs. 66.2 %, respectively, *p* >0.05).

**Conclusions:**

While NGS screening has been recently introduced to assist IVF patients, this is the first randomized clinical study on the efficiency of NGS for preimplantation genetic screening in comparison to aCGH. With the observed high accuracy of 24-chromosome diagnosis and the resulting high ongoing pregnancy and implantation rates, NGS has demonstrated an efficient, robust high-throughput technology for PGS.

## Background

Numerical chromosome abnormality or aneuploidy is the main cause for embryo arrest, implantation failure, recurrent pregnancy loss and birth defects [1–6]. Aneuploidy rate is extremely high in IVF patients, especially in those with recurrent pregnancy loss [4], repeated implantation failure [5] and previous aneuploid conceptions [6]. Aneuploidy is the most common abnormality in in-vitro fertilized zygotes and embryos [7–9], and aneuploidy rate increases with maternal age [7–11]. Selection of chromosomally normal embryos for transfer by aneuploidy screening has been a primary focus of investigation since the inception of PGS [12, 13]. As the field evolved, an increasing number of studies have concentrated on developing more advanced technologies for screening embryos from IVF-PGS treatment cycles in order to eliminate aneuploid embryos and to select euploid embryos for transfer.

Fluorescence in situ hybridization (FISH) was the original method for aneuploidy screening of oocytes and embryos from IVF treatment cycles [12–15]. In early PGS studies, only a limited number (5–12) of chromosomes were screened using FISH, which had an error rate of 5-15 % and resulted in disappointing pregnancy outcomes [16–19]. Conventional comparative genomic hybridization (CGH) was then applied to screen all 24 chromosomes of oocytes and embryos with some success, although it typically took several days to complete CGH testing [20]. Array comparative genomic hybridization has been recently proven to be a reliable method for preimplantation genetic screening within 24 h and has been widely applied in IVF-PGS treatment cycles worldwide [21–31]. Meanwhile, single nucleotide polymorphism (SNP) array [32–35] and qPCR-based comprehensive chromosomal screening (CCS) [7, 36–39] have also been used for screening embryos before transfer in order to improve the efficiency of IVF and PGS treatments. More recently, next-generation sequencing has been introduced into IVF field [40, 41]. With known advantages of robust high-throughput and customizable parallel analysis of multiple samples in a single sequencing run, several NGS platforms have been validated and/or evaluated for preimplantation genetic diagnosis (PGD) of specific mutations of nuclear [42] and mitochondrial genomes [43], and preimplantation genetic screening of chromosomal aberrations [43–46]. However, there is still very limited information about efficiency of NGS-based comprehensive chromosomal screening in terms of clinical pregnancy and implantation outcomes for IVF-PGS patients in a randomized study.

To date, there is no consensus on the best way to screen embryos from IVF-PGS treatment cycles and to select the most competent embryos for transfer despite the recent advances in molecular cytogenetic technologies [40, 47]. Accordingly, our present study aimed at investigating the effects of NGS aneuploidy screening on clinical pregnancy and implantation outcomes for IVF-PGS patients in comparison to the highly validated method of aneuploidy screening, aCGH.

## Methods

### Ethics statement

We obtained ethics approval for our study from the ethics committees (also known as an Institutional Review Board, IRB) at our respective institutions (e.g. ZytoGen's IRB committee). All the participants had the capacity to consent and we obtained the written informed consents from all patients enrolled in the present study.

### Study design and overview

This study was designated and performed in two phases. Phase I study evaluated the accuracy of NGS for aneuploidy screening across all 24 chromosomes in comparison to aCGH. A total of 164 whole-genome amplification (WGA) products were selected from 38 IVF-PGS patients at mean age 35.2 ± 3.4 years whose biopsy and aCGH were performed previously [25, 48]. The PGS indications for this group of patients included: 1) recurrent pregnancy loss (n = 15), 2) repeated implantation failure (n = 13), and 3) previous aneuploidy conceptions (n = 10). These patients had their embryos screened with aCGH and transfer of euploid blastocysts had resulted in ongoing pregnancies and/or live birth of euploid babies as reported previously [25, 48]. The selected WGA products were retrospectively analyzed with NGS and the results were compared with those of aCGH. The accuracy of NGS screening was evaluated by comparing with aCGH results from the overall diagnosis of ploidy in individual blastocysts. In particular, the sensitivity, specificity, positive and negative predictive values of the NGS screening were calculated as described by Fiorentino et al. [45].

Phase II study further compared the clinical pregnancy, ongoing pregnancy and implantation outcomes between NGS (Group A) and aCGH (Group B) for preimplantation genetic screening through a prospective randomized design. PGS patients who met the inclusion criteria were randomized into two groups by using a randomized table: 1) NGS (Group A), PGS patients (n = 86) had their embryos screened with NGS and 2) aCGH (Group B), PGS patients (n = 86) had their embryos screened with aCGH. For both groups, all enrolled patients underwent oocyte retrieval per routine and MII oocytes were fertilized by intra cytoplasmic sperm injection (ICSI). All injected MII oocytes were cultured to blastocyst stage in a time-lapse system as described previously [48]. Blastocysts were vitrified after trophectoderm biopsy. All WGA products of the biopsy samples were analyzed with either NGS (Group A) or aCGH (Group B). One to two euploid blastocysts were thawed and transferred to individual patients primarily based on the PGS results. Clinical pregnancy, ongoing pregnancy and implantation rates were compared between the two study groups.

### Patient’s inclusion criteria and randomization

In Phase II study, a total of 172 patients at mean age 35.2 ± 3.5 years undergoing preimplantation genetic screening were enrolled in this prospective, single-blind, pilot interventional study in our multiple IVF clinics in USA and China from July 2013 to June 2014. A written informed consent was obtained from all patients and pre-treatment counseling was provided to each couple. Standard clinical protocols and laboratory procedures were used for the treatment of all patients in this pilot study. The cohort patients requested PGS due to recurrent pregnancy loss (n = 72), repeated implantation failure (n = 63) and previous aneuploid conceptions (n = 37). The inclusion criteria for enrollment in this study were: 1) female patient’s age ≤ 39 years; 2) ≥ 4 blastocysts available for biopsy on day 5 (up to 11:00 pm); 3) presence of both ovaries and normal uterine lining; 4) undergoing preimplantation genetic screening for their embryos; and 5) willingness to participate in the study and to follow instructions. The exclusion criteria were: 1) patients whose treatment incorporated donor gametes; 2) patients with severe endometriosis; 3) patients with endometrial factors related infertility. A random number table generated by a computer program was used to determine patients in PGS method with either NGS (Group A) or aCGH (Group B). Patients (but not the lab personnel) were blinded with regard to their randomization groups. The two study groups were mutually exclusive, and no study patient had embryos assigned to both study groups.

The randomization table was generated using GraphPad InStat version 3.10 (GraphPad Software, San Diego, California, USA). A block randomization table was typically created for each of three groups with different clinical indications: 1) recurrent pregnancy loss, 2) repeated implantation failure and 3) previous aneuploid conceptions. Block randomization assured that parity of distribution was attained within each block for each clinical indication. Based on the randomization table, a single sheet of paper was prepared for each case indicating whether that number was a Group A (NGS) patient or a Group B (aCGH) patient. The number sheets were placed into sealed envelopes with individual patient numbers. Once a patient was qualified for the study, two lab personnel on duty opened the envelopes and assigned the individual patients into Group A or Group B by following the assigned number. Laboratory supervisors in charge confirmed the assignment of randomization for individual patients in each group. The following materials and methods, unless specified, were applied to phase II study.

### Ovarian stimulation, oocyte retrieval and fertilization

All enrolled female patients had an ultrasound scan and serum evaluation of follicle stimulation hormone (FSH), anti Müllerian hormone (AMH) and estradiol (E_2_) on day 3 of their menses. The patients were then stimulated with conventional down-regulation protocols as described previously [25, 48]. For female patients, oocyte retrieval was performed under transvaginal ultrasound guidance at 35 to 36 h after administration of hCG. After stripping of cumulus cells, oocytes at MII stage were inseminated with ICSI 4 h after retrieval as previously described [25, 48]. Fertilization was assessed 16–18 h post ICSI. For male patients, sperm concentration and motility were evaluated after 3 days of sexual abstinence according to the World Health Organization (WHO) recommendations [49].

### Embryo culture and trophectoderm biopsy

For both study groups, all embryos were cultured from one-cell to blastocyst stage in a continuous single culture medium (CSC, Irvine Scientific, Irvine, USA) plus 12 % synthetic serum substitute (SSS) within a time-lapse system (EmbryoScope™, Unisense FertiliTech, Aarhus, Denmark) at 37 °C, 6 % CO_2_, 5 % O_2_ as described previously [48]. When embryos developed to the blastocyst stage on days 5 (up to 11:00 pm), an opening of 6 to 9 um was made in the zona pellucida with several pulses of 18 ms from a non-contact 1.48 um diode Octax laser system (MTG, Bruckberg, Germany), and 3 to 5 trophectoderm (TE) cells were aspirated into a biopsy pipette and separated from the blastocysts by applying multiple laser pulses of 14 ms between the trophectoderm cells at the stretching area. The biopsied TE cells were washed in 1× PBS and loaded into a PCR tube containing 2.5 μl 1× PBS. All the biopsy and manipulation procedures were performed in a fully enclosed workstation to provide a controlled environment for manipulation of biopsied embryos (Origio, Mt. Laurel, USA) as described previously [48].

### aCGH testing

Whole genomic amplification (WGA) of the biopsy samples and aCGH testing in the aCGH group were performed with the use of the Sureplex DNA Amplification System (BlueGnome, Cambridge, UK) as reported elsewhere [22, 25]. One nanogram of genomic DNA and one reagent-negative control were also subjected to WGA. The WGA products (sample and control DNA) were labeled with Cy3 and Cy5 fluorophores for 2–4 h. Labeled DNA was then resuspended in a dexsulphate hybridization buffer and hybridized onto the 24sure chips under cover slides for 4–6 h. After washing and drying, the hybridized 24sure chips were scanned at 10 μm using a laser scanner (Agilent, Sainte Rosa, USA). The scanning data were then analyzed and quantified by algorithm fixed settings in BlueFuse Multi Software (BlueGnome, Cambridge, UK), a software package that performed the steps of grid placement, quantification, normalization and post-processing automatically. Once a specific amplification was observed (i.e. low autosomal noise), autosomal profiles were analyzed for gain or loss of whole chromosomal ratios using a 3 × SD assessment, greater than ± 0.3 log2 ratio call, or both according to the manufacturer’s instructions (available at www.cytochip.com). To ensure hybridization quality controls, female samples hybridized with a male reference DNA (sex mismatch) had to show a consistent gain on chromosome X and a consistent loss of chromosome Y [22].

#### Sequencing and sequence analysis

**Fig. 1:**
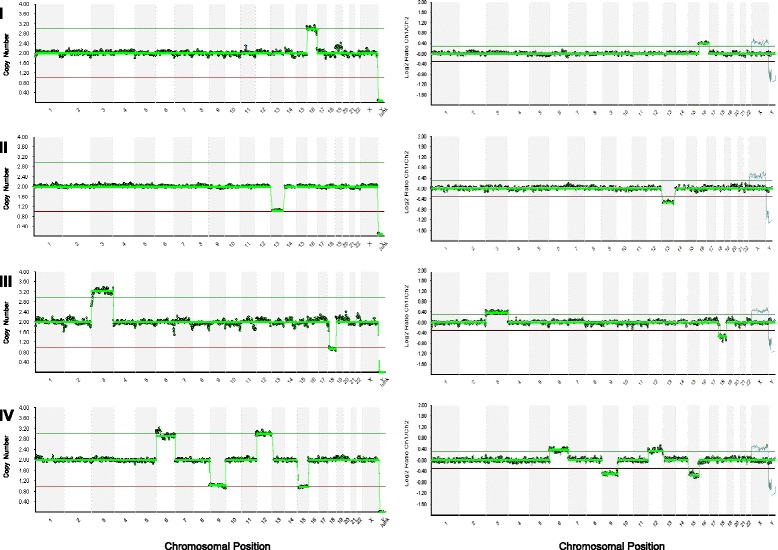
Representative profiles showing different types of aneuploidies detected by NGS (the left panel) and aCGH (the right panel) screening of the same whole genomic amplification (WGA) products. Each NGS profile in the left panel indicates the chromosome numbers on the x-axis and copy numbers of chromosomes on the y-axis. Each aCGH profile in the right panal indicates the chromosome numbers on the x-axis and log ratio of chromosomes on the y-axis. I. Aneuploid profile with single chromosomal gain (trisomy): a gain of chromosome 16; II. Aneuploid profile with single chromosomal loss (monosomy): a loss of chromosome 13; III. Aneuploid profile with dual chromosomal abnormalities: a gain of chromosomes 3 and a loss of 18; IV. Aneuploid profile with complex chromosomal abnormalities: gains of chromosomes 6 and 12 and losses of chromosomes 9 and 15

**Fig. 2:**
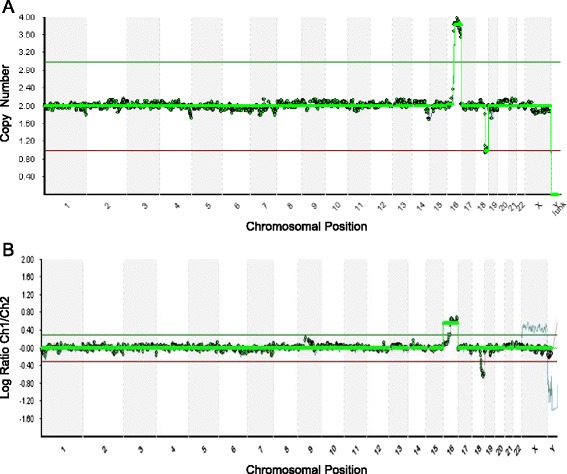
Representative profiles showing segmental imbalances detected by NGS (the upper profile) and aCGH (the lower profile) screening of the same WGA product. The upper profile (**a**) was resulted from NGS screening which revealed a 42 Mb gain on the q arm of chromosome 16 and a 16 Mb loss on the q arm of chromosome 18 more precisely compared to aCGH screening in the lower profile (**b**)

**Fig. 3:**
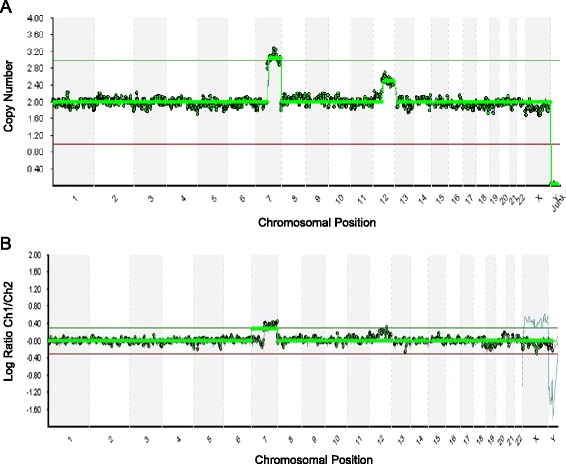
Representative profiles showing mosaicism resulted from NGS (the upper profile) and aCGH (the lower profile) screening of the same WGA product. The upper profile (**a**) was resulted from NGS screening which revealed a 46 % mosaicism of chromosome 12 accurately. The lower profile (**b**) was resulted from aCGH screening of the same WGA product, which was unable to detect the mosaicism of chromosome 12

Whole genomic amplification of the biopsy samples in the NGS group was performed using the same method as the aCGH group. WGA products were then quantified using the QuanTit dsDNA HS Assay Kit (Life Technologies Corporation, Grand Island, NY, USA). Dual-indexed libraries were prepared using the Nextera XT DNA Sample Preparation Kit and Index Kits with the input sample DNA at 0.2 ng/μl (1 ng total) (Illumina, San Diego, USA). The quality of a subset of libraries was assessed using the Agilent High Sensitivity DNA Kit (Agilent Technologies Inc, Santa Clara, CA, USA) and by sequencing with the MiSeq Reagent Kit v3 (Illumina Inc., San Diego, USA). Paired-end, dual index 2x36bp sequencing was performed using the Illumina workflow on a HiSeq 2000 with 96-plex per lane (Illumina, San Diego, USA). Reads were aligned to the human genome hg19 using *iSAAC* within the HiSeq Analysis Software. Bash scripting, BEDtools and SAMtools were used to remove unmapped reads, duplicate reads, reads with low mapping scores and reads with an edit distance greater than one. Each chromosome was divided into intervals each approximately covering 1 Mb of sequence. Filtered reads from each sample were then mapped into the corresponding chromosome interval or bin. The count data in each bin was normalized using GC content, and *in-silico* reference data in order to remove bias. The normalized bin counts were then re-expressed as copy number by assuming the median autosomal read count corresponds to copy number two. The bin-wise copy number values for each chromosome were smoothed with a 13-bin sliding median. Automated copy-number status for each chromosome was determined using the median of smoothed copy-number values across the chromosome as described elsewhere [45]. In particular, the analysis pipeline expected a default copy number of 2 for autosomes; the sample sex and sex chromosome copy numbers were determined by an initial calling algorithm. Embryos were diagnosed as abnormal or aneuploid if the median chromosomal copy number measures deviated from the default copy number. Chromosomal gain or trisomy (copy number >2) and chromosomal loss or monosomy (copy number <2) are seen as horizontal green bars above and below, respectively in Figs. 1, 2 and 3, the copy number state of 2. The method also allows a specific copy number (1, 2, 3, or 4) to be directly assigned. Embryos were diagnosed as normal or euploid if the generated plot showed no gain or loss.

### Blastocyst vitrification and warming

After trophectoderm biopsy, blastocysts were vitrified using the Cryotip method as previously described [26]. In brief, blastocysts were equilibrated in equilibration solution (ES) containing 7.5 % DMSO, 7.5 % ethyleneglycol and 20 % SSS for 10–12 min. They were then passed through four 20 μl drops of virification solution (VS) containing 15 % DMSO, 15 % ethyleneglycol, 0.5 M sucrose and 20 % SSS. Individual blastocysts were loaded into the Cryotips within 90 s and plunged into liquid nitrogen immediately. For warming, the Cryotip containing embryos was removed from liquid nitrogen and thawed in a 37 °C water bath for about 3 s and the contents were released as a small drop in a culture dish. The Cryotip contents were then mixed with the thawing solution (TS) containing 1.0 M sucrose, 20 % SSS in modified human tubal fluid (mHTF) for 1 min. The blastocysts were passed through two drops of dilution solution (DS) containing 0.5 M sucrose, 20 % SSS. They were then passed through two drops of washing solution (WS) containing 20 % SSS in mHTF before placing into blastocyst culture medium. After warming, one to two euploid blastocysts were transferred to each patient depending on individual patient’s age and indications, as well as survival of the vitrified euploid blastocysts available. No more than two blastocysts were transferred to each patient in order to avoid high-order multiple pregnancies.

### Embryo assessment and transfer

In both NGS and aCGH groups, fertilization was assessed at 16 to 18 h post ICSI and the fertilized zygotes were then cultured to blastocyst stage in the time-lapse system as described previously [48]. Images of individual embryos were captured with a built-in digital camera every 20 min at 7 different focal planes. Fertilization was assessed at 16 to 18 h post ICSI insemination according to the digital images acquired with the time-lapse monitoring system. Detailed analysis of the acquired images of each embryo was made with the EmbryoView software (Unisense FertliTech, Denmark), and all the targeted events related to embryonic development were then annotated together with the corresponding hour(s) post ICSI insemination (hpi). All morphokinetic data were recorded as mean ± SD hpi.

Selection of embryos for transfer was primarily based on PGS results with NGS (Group A) or aCGH (Group B). When multiple euploid blastocysts were recognized from individual patients, the morphokinetic markers were the secondary criterion for selection according to the most predictive parameters that were highly correlated with implantation as described elsewhere [50, 51]. One to two euploid blastocysts within the most predictive parameters available were selected for transfer to individual patients after warming. For both study groups, patients were treated using identical endometrial preparation protocols as previously reported [26].

### Sample size calculation and statistical analysis

Sample size in Phase II study was calculated using GraphPad StatMate (GraphPad Software, San Diego, California, USA). Based on our previous clinical studies in which about 42 % of all transferred blastocysts implanted after transfer [25, 26, 48], a minimum sample size of 110 blastocysts for transfer in each group had an 80 % power to detect a difference between means of 0.20 with a significance level of 0.05 (two-tailed value). The percentages of euploid and aneuploid blastocysts were recorded and compared between NGS (Group A) and aCGH (Group B). Clinical pregnancy, implantation and ongoing pregnancy rates were also tabulated and compared between the two study groups. Clinical pregnancy was defined as an intrauterine gestational sac with fetal heartbeat visualized by ultrasound examination at week 8 after embryo transfer. Ongoing pregnancy was defined as continuing pregnancy at ≥ 20 weeks of gestation. Implantation rate was calculated as the total number of sacs with fetal hearts beat over the total embryos transferred. The categorical variables were analyzed by Chi-square analysis or Fisher’s exact test as appropriate. The numerical parameters were analyzed with *t*-test. The time-lapse variables were first tested for normality using the Shapiro-Wilk test and then analyzed by the Mann–Whitney test. The statistical analyses were performed using GraphPad InStat version 3.10 (GraphPad Software, San Diego, USA). A two-tailed value of *p* <0.05 was considered statistically significant.

## Results

### Results from phase I study

A total of 164 WGA products derived from 38 IVF-PGS treatment cycles were analyzed with NGS in comparison to aCGH. The testing results from the same WGA products were compared between the two methods. A total of 3936 (164 x 24) chromosomes from the biopsied blastocysts (n = 164) were assessed for the entire copy number gains (copy number > 2) and losses (copy number < 2). NGS specificity for aneuploidy blastocyst call (all 24 chromosome diagnosis consistency) was 100 % (95 % CI: 95.32 %–100 %) with a sensitivity of a 100 % (95 % CI: 98.16 %–100 %). Both positive and negative predictive values of the NGS screening were 100 % (Table 1).

**Table 1:** NGS technical assessment as compared to aCGH in Phase I study

Parameters	Results
Total number of blastocysts analyzed	164
Number of euploid blastocysts (true negative)	61
Number aneuploid blastocysts (true positive)	103
Number of missed aneuploid blastocyst call (false negative)	0
Number of extra aneuploidy blastocyst call (false positive)	0
Aneuploid blastocyst call specificity % (95 % CI)	100 % (95.32-100 %)
Aneuploid blastocyst call sensitivity % (95 % CI)	100 % (98.16-100 %)
Positive predictive value % (95 % CI)	100 % (97.43-100 %)
Negative predict value % (95 % CI)	100 % (95.25-100 %)

*CI* confidence interval, *Specificity* true negatives/(true negatives + false positives), *Sensitivity* true positives/(true positives + false negatives), *Positive predictive value* true positives/(true positives + false positives), *Negative predictive value* true negatives / (false negatives + true negatives)

NGS detected all types of aneuploidies including single chromosome gain (or trisomy), single chromosome loss (or monosomy), dual (two) and complex (three or more) chromosomal abnormalities accurately compared to aCGH (Fig. 1). NGS provided a 100 % 24-chromosome diagnosis (of euploid and aneuploid) consistency with the highly validated method for aneuploidy screening, aCGH (Table 2).

**Table 2:** Comparison of percentages of different types of aneuplodies detected by NGS and aCGH screening of the same WGA products in Phase I study

Parameters	NGS	aCGH	*p*
Total number of WGA products analyzed	164	164	
Euploid % (n)	37.2 % (61)	37.2 % (61)	1.000^a^
Aneuploid % (n)	62.8 % (103)	62.8 % (103)	1.000^a^
Monosomy % (n)	19.4 % (20)	19.4 % (20)	1.000^a^
Trisomy % (n)	16.5 % (17)	16.5 % (17)	1.000^a^
Dual chromosomal abnormality % (n)	24.3 % (25)	24.3 % (25)	1.000^a^
Complex chromosomal abnormality % (n)	35.9 % (37)	37.8 % (39)	0.885^a^
Mosaicism % (n)	2.5 % (4)	1.2 % (2)	0.683^b^

^a^by Chi-square analysis

^b^by Fisher’s exact test

Detailed comparison of partial aneuploidies detected by NGS and aCGH screening of the same WGA product revealed that NGS detected partial chromosomal gains and losses more precisely (Fig. 2), suggesting that NGS may detect aneuploidy and segmental imbalances at the same time. Further comparison of mosaicism detected by NGS and aCGH screening of the same WGA product revealed that NGS provided more accurate detection of mosaicism of the trophectoderm cells from blastocyst biopsy (Fig. 3).

### Results from phase II study

**Fig. 4:**
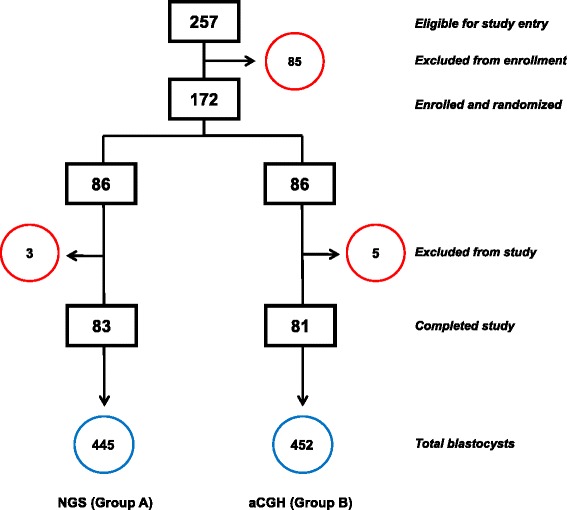
Schematic for IVF-PGS patients randomized into either NGS (Group **a**) or aCGH (Group **b**). Excluded patients in each group were circled in red. The total number of blastocysts associated with each study group is circled in blue

As shown in Fig. 4, 257 patients were eligible for the study entry and 85 of them were excluded from enrollment due to personal reasons (n = 38), financial difficulties (n = 26) or medical complications (n = 21). A total of 172 patients at mean age 35.2 ± 3.5 years (ranging from 28 to 39 years old) who met the inclusion criteria were randomized into either NGS (Group A, n = 86) or aCGH (Group B, n = 86). Of these, 3 patients in the NGS group and 5 patients in the aCGH group withdrew from the treatments for medical reasons. For Group A and Group B, 83 and 81 patients completed the study and were included in the final data analysis respectively. The demographic parameters of female and male patients were comparable between the two groups (Table 3). There were no significant differences in female patient’s mean age, day 3 FSH, AMH, E_2_, antral follicle number, male patient’s sperm count and motility between the two groups (*p* >0.05). As shown in Table 4, there were no significant differences in fertilization rate (per MII oocytes) between Group A and Group B (89.8 % vs. 88.7 %, respectively, *p* >0.05). Moreover, the blastocyst formation rate (per MII oocytes) in Group A was also similar to that of Group B (48.9 % vs. 49.8 %, respectively, *p >*0.05).

**Table 3:** Comparison of patient’s demographic parameters between NGS (Group A) and aCGH (Group B) in Phase II study

Parameters	NGS	aCGH	*p*
Total number of patients completed study	83	81	
Mean female age ± SD	35.5 ± 3.3	35.2 ± 3.5	0.573
Mean D3 FSH (mUI/mL) ± SD	7.2 ± 1.6	7.1 ± 1.5	0.680
Mean AMH (ng/mL) ± SD	5.1 ± 2.1	4.6 ± 2.3	0.148
Mean E_2_ (pg/mL) ± SD	25.7 ± 3.2	26.3 ± 3.6	0.261
Mean antral follicles ± SD	12.3 ± 3.0	12.8 ± 2.8	0.272
Mean sperm count (million/mL) ± SD	35.4 ± 4.5	36.1 ± 4.2	0.305
Mean sperm motility (%) ± SD	49.6 ± 15.1	48.5 ± 14.7	0.637

There is no significant difference in any of the parameters between the two groups (*p* >0.05, by *t* test)

*SD* Standard deviation, *NS* No significant difference between the two groups, *FSH* Follicle stimulation hormone, *AMH* Anti-Müllerian hormone, *E*_*2*_ Estradiol

**Table 4:** Comparison of fertilization and blastocyst formation rates between NGS (Group A) and aCGH (Group B) in Phase II study

Parameters	NGS	aCGH	*p*
Total number of oocytes retrieved	1063	1049	
MII oocytes % (n)	85.9 % (914)	86.4 % (906)	0.847^a^
Oocytes fertilized (2PN) % (n)	89.8 % (821)	88.7 % (804)	0.502^a^
Blastocysts % (n)	48.9 % (447)	49.8 % (452)	0.709^a^

*MII* metaphase II, *2PN* two pronuclei

^a^by Chi-square analysis

As summarized in Table 5, a total of 418 (93.5 %) blastocysts in Group A were biopsied and analyzed by NGS. Biopsies could not be completed for 29 (6.5 %) blastocysts due to poor morphology or they degenerated after biopsy. NGS analysis revealed euploidy in 163 (38.9 %) and aneuploidy in 249 (59.6 %) of the biopsied blastocysts. No signals occurred in 6 (1.4 %) of the biopsied blastocysts due to DNA amplification failure. In Group B, a total of 427 (94.5 %) blastocysts were biopsied while 25 (5.5 %) blastocysts were not biopsied because of poor morphology or they degenerated after biopsy. aCGH analysis revealed euploidy in 171 (40.0 %), aneuploidy in 247 (57.8 %) and no signals in 9 (2.1 %) of the biopsied blastocysyts. Moreover, chromosomal abnormalities were detected across all 24 chromosomes in both groups. As shown in Table 6, all types of aneuploidies were observed in both Group A and Group B, including trisomy, monosomy, dual and complex chromosomal abnormalities. There were no significant differences in the proportions of each type of aneuploidy between the two study groups (*p* >0.05).

**Table 5:** Comparison of biopsy and screening results between NGS (Group A) and aCGH (Group B) in Phase II study

Parameters	NGS	aCGH	*p*
Total number of blastocysts	447	452	
Biopsied blastocysts % (n)	93.5 % (418)	94.5 % (427)	0.643^a^
Euploid % (n)	38.9 % (163)	40.0 % (171)	0.809^a^
Aneuploid % (n)	59.6 % (249)	57.8 % (247)	0.661^a^
No signal % (n)	1.4 % (6)	2.1 % (9)	0.604^b^

^a^by Chi-square analysis

^b^by Fisher’s exact test

**Table 6:** Comparison of screening results of each type of aneuploid blastocysts between NGS (Group A) and aCGH (Group B) in Phase II study

Parameters	NGS	aCGH	*p*
Total number of aneuploid blastocysts	249	247	
Monosomy % (n)	21.7 % (54)	21.1 % (52)	0.950^a^
Trisomy % (n)	16.5 % (41)	17.8 % (44)	0.780^a^
Dual chromosomal abnormality % (n)	22.1 % (55)	23.1 % (57)	0.876^a^
Complex chromosomal abnormality % (n)	36.9 % (92)	36.8 % (91)	0.981^a^
Mosaicism % (n)	2.8 % (7)	1.2 % (3)	0.339^b^

^a^by Chi-square analysis

^b^by Fisher’s exact test

**Fig. 5:**
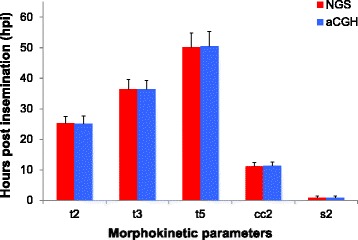
Comparison of morphokinetic parameters of the early stages of embryonic development between NGS (red) and aCGH (blue) groups. t2 = time from insemination to 2 cells; t3 = time from insemination to 3 cells; t5 = time from insemination to 5 cells; cc2 = time between division to 2 cells and division to 3 cells; s2 = time between division to 3 cells and subsequent division to 4 cells; hpi = hours post insemination. Morphokinetic data were presented as mean ± SD. There were no significant differences in each of the morphokinetic parameters between NGS and aCGH groups (*p* >0.05, by Mann–Whitney test)

The most predictive morphokinetic parameters that were highly correlated with implantation [50, 51] were compared between the two study groups (Fig. 5). There were no significant differences in the time from insemination to 5 cells (t5) between Group A and Group B (50.2 ± 4.5 hpi vs. 50.4 ± 4.7 hpi, respectively, *p >*0.05). The time between division to 2 cells and division to 3 cells (cc2) was similar in the two groups (11.3 ± 1.1 hpi vs. 11.2 ± 1.2 hpi, respectively, *p >*0.05). Moreover, the time between division to 3 cells and subsequent division to 4 cells (s2) was also comparable between the two study groups (0.78 ± 0.68 hpi vs. 0.77 ± 0.69 hpi, respectively, *p >*0.05).

For both study groups, one to two euploid blastocysts within the most predictive morphokinetic parameters available were selected for transfer to individual patients. As summarized in Table 7, a total of 79 (95.2 %) patients had euploid blastocyst(s) for transfer while 4 (4.8 %) of them ended with no euploid embryos available for transfer in Group A. Among the patients with euploid blastocysts for transfer, 27 patients had single euploid blastocysts and 52 patients had double euploid blastocysts for transfer. In Group B, a total of 78 (96.3 %) patients had euploid blastocysts for transfer while 3 (3.7 %) of the patients ended with no euploid embryos available for transfer. Of the patients with embryo transfer, 23 patients had single euploid blastocysts and 55 had double euploid blastocysts for transfer. There was no significant difference in clinical pregnancy rate between Group A and Group B (75.9 % vs. 71.8 %, respectively, *p* >0.05). The observed implantation rate in Group A was similar to that of Group B (70.5 % vs. 66.2 %, respectively, *p* >0.05). Ongoing pregnancy rate was also comparable between Group A and Group B (74.7 % vs. 69.2 %, respectively, *p* >0.05). Additionally, there was no significant difference in miscarriage rate between NGS and aCGH (1.3 % vs. 2.6 %, respectively, *p >*0.05).

**Table 7:** Comparison of pregnancy and implantation outcomes between NGS (Group A) and aCGH (Group B) in Phase II study

Parameters	NGS	aCGH	*p*
Patients with SET	27	23	
Patients with DET	52	55	
Clinical pregnancy rate with SET % (n)	62.9 % (17)	60.9 % (14)	0.879^a^
Clinical pregnancies rate with DET % (n)	82.2 % (43)	76.4 % (42)	0.568^a^
Overall clinical pregnancy rate % (n)	75.9 % (60)	71.8 % (56)	0.681^a^
Overall implantation rate % (n)	70.5 % (92)	66.2 % (88)	0.564^a^
Overall ongoing pregnancy rate % (n)	74.7 % (59)	69.2 % (54)	0.560^a^
Overall miscarriage rate % (n)	1.3 % (1)	2.6 % (2)	0.620^b^

*SET* single embryo transfer, *DET* double embryo transfer

^a^by Chi-square analysis

^b^by Fisher’s exact test

## Discussion

The ultimate goal of preimplantation genetic screening of oocytes and embryos from in vitro fertilization treatments is to select one to two chromosomally normal embryos for transfer, so as to maximize the chances of successful pregnancies and to minimize the incidences of harmful miscarriages. As discussed previously, aneuploidy rate is extremely high in IVF patients with recurrent pregnancy loss, repeated implantation failure and previous aneuploid conceptions [4–6]. Early randomized clinical trials with FISH screening of a limited numbers of chromosomes resulted in disappointing pregnancy outcomes [16–19]. Recent studies with aCGH screening of 24 chromosomes have resulted in a significant increase in clinical and ongoing pregnancy rates for patients either seeking single embryo transfer (SET) [25, 26] or undergoing preimplantation genetic screening [21–24, 27–31, 48]. More recent studies using cells of known genetic complements and/or WGA products derived from blastomere biopsy have indicated that NGS is highly sensitive for aneuploidy screening [45], and transfer of the screened embryos has resulted in viable pregnancies in several case studies [43, 44, 46]. In our randomized pilot study, we provided further clinical evidence demonstrating that NGS screening has resulted in similarly high ongoing pregnancy and implantation rates compared to aCGH screening. To date, this is the first randomized clinical study on the efficiency of NGS-based comprehensive chromosomal screening for IVF-PGS patients in comparison to aCGH-based comprehensive chromosomal screening.

In the present study, the accuracy and efficiency of NGS screening of all 24 chromosomes were measured in two phases. Phase I study evaluated accuracy of NGS screening of WGA products selected from previously performed IVF-PGS cycles in our IVF clinics. We chose the NGS and aCGH platforms that were developed and produced by the same manufacturer (Illumina, San Diego, USA) for strict comparison purposes. The aCGH platform used in this study is the first technology that has been widely adopted for aneuploidy screening across all 24 chromosomes and has been extensively used in IVF-PGS treatments worldwide [21–31]. The NGS platform used in this study has been recently validated by using cells of known abnormal genetic complements and WGA products derived from blastomere biopsy of cleavage-stage embryos [45]. The present study, using trophectoderm cells from blastocyst biopsy, has provided further clinical evidence showing that the NGS screening accurately detected all types of aneuploidies of human blastocysts compared to aCGH screening. Our current data also confirmed previous observations [45, 46] that NGS provided a 100 % 24-chromosome diagnosis consistency with the highly validated aCGH method. In particular, all embryos (n =61) diagnosed as euploid by NGS were proven to be euploid with aCGH and all embryos (n =103) diagnosed as aneuploidy by NGS were confirmed as aneuploid by aCGH. Moreover, the NGS platform presented here has also shown a capability of detecting segmental changes more precisely compared to aCGH, suggesting that the detection of partial aneuploidies or imbalanced translocations is feasible with the use of this NGS platform. Additionally, comparison of mosaicism detected by NGS and aCGH screening of the same WGA product revealed that NGS provided more accurate detection of mosaicism compared to aCGH.

There were additional advantages with use of NGS screening compared to aCGH screening although the current cost ($120 to $130 per sample) and the turn-around time (18 to 20 h) for 24-chromosome screening were similar between the two methods. With the use of DNA barcoding technologies, NGS screening offers a unique method for evaluation of multiple samples from multiple patients with different indications, such as recurrent pregnancy loss, repeated implantation failure and previous aneuploid conceptions, on the same sequencing chip. Up to 96 samples can be analyzed in a single run with the use of an upgraded NGS instrument with high capacity (e.g. Hiseq). In addition, NGS screening does not require co-hybridization of DNA control samples while aCGH testing requires both male and female DNA controls in the hybridization step. With increasing volume of PGS cases worldwide and improvement of platform production on a larger scale, it is predictable that NGS method may ultimately lead to lower costs per patient in the near future.

Phase II study further compared the clinical pregnancy and implantation outcomes between NGS (Group A) and aCGH (Group B) for preimplantation genetic screening. A large number (n = 172) of IVF-PGS patients who meet the inclusion criteria were randomized into the two study groups. Data analysis showed that the demographic parameters of both female and male patients in the two study groups were similar (*p* >0.05). The fertilization and blastocyst rates were also comparable between the two study groups (*p* >0.05). The parallel comparison of the results from transfer of the screened blastocysts revealed that there were no significant differences in the clinical pregnancy, ongoing pregnancy and implantation rates between NGS and aCGH groups (*p* >0.05). Collectively, our pilot data clearly demonstrate that NGS is an efficient, robust technology for comprehensive chromosomal screening for IVF-PGS patients compared to the clinically proven technology, aCGH. Recent studies with NGS screening of embryos from several series of IVF cases at a small scale have also resulted in viable pregnancies [43–46]. Our current data represent the first randomized clinical study with a larger group of IVF-PGS patients to investigate the effects of NGS screening on clinical pregnancy and implantation outcomes in comparison to aCGH screening.

It is worth mentioning that all embryos in both Group A and Group B throughout Phase II study were cultured and monitored in the time-lapse system in order to maximize the chances of a successful pregnancy. Our previous study with sibling oocytes demonstrated that the combination of time-lapse monitoring and aCGH screening resulted in high clinical pregnancy and implantation rates for IVF-PGS patients [48]. By using the same approach, embryos in the present study were screened with either NGS (Group A) or aCGH (Group B) before transfer, and selection of euploid blastocyst(s) for transfer was primarily based on PGS results for both groups. When there were multiple euploid blastocysts available for transfer in each patient, the most predictive morphokinetic parameters were the secondary criterion for embryo selection. Our present study has contributed new clinical data demonstrating that the combination of time-lapse monitoring and NGS screening has resulted in similarly high clinical pregnancy, ongoing pregnancy and implantation rates for PGS patients compared to the combined use of time-lapse monitoring and aCGH screening.

Several limitations of our pilot study should be addressed. First, although NGS-based comprehensive chromosomal screening brings distinct benefits for many IVF-PGS patients, the approach is not for all patients, especially for those with diminished ovarian reserve. The improved pregnancy rates achieved here may not be fully applied to all IVF-PGS patients, especially those at advanced maternal age of 40 years or above. Although it is possible to accumulate enough blastocysts from multiple IVF treatment cycles by vitrification [52] before NGS screening of the accumulated embryos from the patients with diminished ovarian reserve or at advanced maternal age, further randomized clinical trials with a larger sample are required to confirm its clinical benefits for these patients. Second, similar to other molecular cytogenetic technologies, NGS is unable to detect balanced chromosomal rearrangements directly due to lack of imbalance in the total DNA content. Moreover, our data showed that there was no significant difference in mosaicism rate between NGS and aCGH groups (2.8 % vs. 1.2 %, respectively, *p* >0.05). This may be due to insufficient patient numbers in this category or limited numbers of trophectoderm cells (3–5) being analyzed in each biopsy sample. Finally, the WGA products in the present study were not defined for unbalanced translocation breakpoints, although our current data was in agreement with previous studies [45, 46] demonstrating that NGS may detect chromosomal aneuploidy and imbalanced derivatives at the same time. Hence, additional studies with the use of cell lines or WGA products from parents who carry known translocation breakpoints are needed in order to assess the accuracy and limits of NGS platforms, with high read depth and/or proper coverage, for detection of imbalanced translocations.

## Conclusion

In this randomized pilot study, we have demonstrated that NGS detects all types of aneuploidies of human blastocysts accurately and provides an extremely high level of 24-chromosome diagnosis consistency with aCGH. Moreover, NGS screening identifies euploid blastocysts for transfer and results in similarly high ongoing pregnancy and implantation rates for IVF-PGS patients compared to aCGH screening. A multi-center randomized clinical trial with a larger sample is planned to define the role of NGS in assisted reproductive medicine.

## References

[1] Alfarawati S, Fragouli E, Colls P, Stevens J, Gutierrez-Mateo C, Schoolcraft WB, Katz-Jaffe MG, Wells D (2011). The relationship between blastocyst morphology, chromosomal abnormality, and embryo gender. Fertil Steril.

[2] Hodes-Wertz B, Grifo J, Ghadir S, Kaplan B, Laskin CA, Glassner M, Munné S (2012). Idiopathic recurrent miscarriage is caused mostly by aneuploid embryos. Fertil Steril.

[3] Wilton L (2005). Preimplantation genetic diagnosis and chromosome analysis of blastomeres using comparative genomic hybridization. Hum Reprod Update.

[4] Rubio C, Simon C, Vidal F, Rodrigo L, Pehlivan T, Remohi J, Pellicer A (2003). Chromosomal abnormalities and embryo development in recurrent miscarriage couples. Hum Reprod.

[5] Voullaire L, Wilton L, McBain J, Callaghan T, Williamson R (2002). Chromosome abnormalities identified by comparative genomic hybridization in embryos from women with repeated implantation failure. Mol Hum Reprod.

[6] Munné S, Sandalinas M, Magli C, Gianaroli L, Cohen J, Warburton D (2004). Increased rate of aneuploid embryos in young women with previous aneuploid conceptions. Prenat Diagn.

[7] Franasiak JM, Forman EJ, Hong KH, Werner MD, Upham KM, Treff NR, Scott RT (2014). The nature of aneuploidy with increasing age of the female partner: a review of 15,169 consecutive trophectoderm biopsies evaluated with comprehensive chromosomal screening. Fert Steril.

[8] Mantzouratou A, Delhanty JDA (2011). Aneuploidy in the human cleavage stage embryo. Cytogenet Genome Res.

[9] Fragouli E, Wells D (2011). Aneuploidy in the human blastocyst. Cytogenet Genome Res.

[10] Munné S, Alikani M, Tomkin G, Grifo J, Cohen J (1995). Embryo morphology, developmental rates, and maternal age are correlated with chromosome abnormalities. Fertil Steril.

[11] Hassold T, Hunt P (2009). Maternal age and chromosomally abnormal pregnancies: what we know and what we wish we knew. Curr Opin Pediatr.

[12] Delhanty JD, Griffin DK, Handyside AH, Harper J, Atkinson GH, Pieters MH, Winston RM (1993). Detection of aneuploidy and chromosomal mosaicism in human embryos during preimplantation sex determination by fluorescent in situ hybridization, (FISH). Hum Mol Genet.

[13] Munne S, Lee A, Rosenwaks Z, Grifo J, Cohen J (1993). Diagnosis of major chromosome aneuploidies in human preimplantation embryos. Hum Reprod.

[14] Harper JC, Delhanty JD (1996). Detection of chromosomal abnormalities in human preimplantation embryos using FISH. J Assist Reprod Genet.

[15] Gianaroli L, Magli MC, Ferraretti AP, Fiorentino A, Garrisi J, Munné S (1997). Preimplantation genetic diagnosis increases the implantation rate in human in vitro fertilization by avoiding the transfer of chromosomally abnormal embryos. Fertil Steril.

[16] Staessen C, Verpoest W, Donoso P, Haentjens P, Van der Elst J, Liebaers I, Devroey P (2008). Preimplantation genetic screening does not improve delivery rate in women under the age of 36 following single-embryo transfer. Hum Reprod.

[17] Hardarson T, Hanson C, Lundin K, Hillensjo T, Nilsson L, Stevic J, Reismer E, Borg K, Wikland M, Bergh C (2008). Preimplantation genetic screening in women of advanced maternal age caused a decrease in clinical pregnancy rate: a randomized controlled trial. Hum Reprod.

[18] Schoolcraft WB, Katz-Jaffe MG, Stevens J, Rawlins M, Munné S (2009). Preimplantation aneuploidy testing for infertile patients of advanced maternal age: a randomized prospective trial. Fertil Steril.

[19] Debrock S, Melotte C, Spiessens C, Peeraer K, Vanneste E, Meeuwis L, Meuleman C, Frijns J-P, Vermeesch JR, D′Hooghe TM (2010). Preimplantation genetic screening for aneuploidy of embryos after in vitro fertilization in women aged at least 35 years: a prospective randomized trial. Fertil Steril.

[20] Wells D, Delhanty JD (2000). Comprehensive chromosomal analysis of human preimplantation embryos using whole genome amplification and single cell comparative genomic hybridization. Mol Hum Reprod.

[21] Fishel S, Gordon A, Lynch C, Dowell K, Ndukwe G, Kelada E, Thornton S, Jenner L, Cater E, Brown A, Garcia-Benardo J (2010). Live birth after polar body array comparative genomic hybridization prediction of embryo ploidy-the future of IVF?. Fertil Steril.

[22] Gutierrez-Mateo C, Colls P, Sanchez-Garcia J, Escudero T, Prates R, Ketterson K, Wells D, Munné S (2011). Validation of microarray comparative genomic hybridization for comprehensive chromosome analysis of embryos. Fertil Steril.

[23] Fiorentino F, Spizzichino L, Bono S, Biricik A, Kokkali G, Rienzi L, Ubaldi FM, Iammarrone E, Gordon A, Pantos K (2011). PGD for reciprocal and Robertsonian translocations using array comparative genomic hybridization. Hum Reprod.

[24] Geraedts J, Montag M, Magli MC, Repping S, Handyside A, Staessen C, Harper J, Schmutzler A, Collins J, Goossens V, van der Ven H, Vesela K, Gianaroli L (2011). Polar body array CGH for prediction of the status of the corresponding oocyte. Part I: clinical results. Hum Reprod.

[25] Yang Z, Liu J, Collins GS, Salem SA, Liu X, Lyle SS, Peck AC, Sills ES, Salem RD (2012). Selection of single blastocysts for fresh transfer via standard morphology assessment alone and with array CGH for good prognosis IVF patients: results from a randomized pilot study. Mol Cytogenet.

[26] Yang Z, Salem SA, Liu X, Kuang Y, Salem RD, Liu J (2013). Selection of euploid blastocysts for cryopreservation with array comparative genomic hybridization (aCGH) results in increased implantation rates in subsequent frozen and thawed embryo transfer cycles. Mol Cytogenet.

[27] Capalbo A, Bono S, Spizzichino L, Biricik A, Baldi M, Colamaria S, Ubaldi FM, Rienzi L, Fiorentino F (2013). Sequential comprehensive chromosome analysis on polar bodies, blastomeres and trophoblast: insights into female meiotic errors and chromosomal segregation in the preimplantation window of embryo development. Hum Reprod.

[28] Rubio C, Rodrigo L, Mir P, Mateu E, Peinado V, Milán M, Al-Asmar N, Campos-Galindo I, Garcia S, Simón C (2013). Use of array comparative genomic hybridization (array-CGH) for embryo assessment: clinical results. Fertil Steril.

[29] Harton GL, Munné S, Surrey M, Grifo J, Kaplan B, McCulloh DH, Griffin DK, Wells D, PGD Practitioners Group (2013). Diminished effect of maternal age on implantation after preimplantation genetic diagnosis with array comparative genomic hybridization. Fertil Steril.

[30] Greco E, Bono S, Ruberti A, Lobascio AM, Greco P, Biricik A, Spizzichino L, Greco A, Tesarik J, Minasi MG, Fiorentino F (2014). Comparative genomic hybridization selection of blastocysts for repeated implantation failure treatment: a pilot study. Biomed Res Int.

[31] Rechitsky S, Pakhalchuk T, Ramos GS, Goodman A, Zlatopolsky Z, Kuliev A (2015). First systematic experience of preimplantation genetic diagnosis for single-gene disorders, and/or preimplantation human leukocyte antigen typing, combined with 24-chromosome aneuploidy testing. Fertil Steril.

[32] Treff NR, Su J, Tao X, Levy B, Scott RT (2010). Accurate single cell 24 chromosome aneuploidy screening using whole genome amplification and single nucleotide polymorphism microarrays. Fertil Steril.

[33] Johnson DS, Gemelos G, Baner J, Ryan A, Cinnioglu C, Banjevic M, Ross R, Alper M, Barrett B, Frederick J, Potter D, Behr B, Rabinowitz M (2010). Preclinical validation of a microarray method for full molecular karyotyping of blastomeres in a 24-h protocol. Hum Reprod.

[34] Lathi RB, Massie JAM, Gilani M, Milki AA, Westphal LM, Baker VL, Behr B (2012). Outcomes of trophectoderm biopsy on cryopreserved blastocysts: a case series. Reprod Biomed Online.

[35] Liu J, Wang W, Sun X, Liu L, Jin H, Li M, Witz C, Williams D, Griffith J, Skorupski J, Haddad G, Gill J (2012). DNA microarray reveals that high proportions of human blastocysts from women of advanced maternal age are aneuploid and mosaic. Biol Reprod.

[36] Scott RT, Ferry K, Su J, Tao X, Scott K, Treff NR (2012). Comprehensive chromosome screening is highly predictive of the reproductive potential of human embryos: a prospective, blinded, nonselection study. Fertil Steril.

[37] Treff NR, Tao X, Ferry KM, Su J, Taylor D, Scott RT (2012). Development and validation of an accurate quantitative real-time polymerase chain reaction-based assay for human blastocyst comprehensive chromosomal aneuploidy screening. Fertil Steril.

[38] Forman EJ, Tao X, Ferry KM, Taylor D, Treff NR, Scott RT (2012). Single embryo transfer with comprehensive chromosome screening results in improved ongoing pregnancy rates and decreased miscarriage rates. Hum Reprod.

[39] Scott RT, Upham KM, Forman EJ, Hong KH, Scott KL, Taylor D, Tao X, Treff NR (2013). Blastocyst biopsy with comprehensive chromosome screening and fresh embryo transfer significantly increases in vitro fertilization implantation and delivery rates: a randomized controlled trial. Fertil Steril.

[40] Handyside AH (2013). 24-chromosome copy number analysis: a comparison of available technologies. Fertl Steril.

[41] Handyside AH, Wells D, Gardner DK, Sakkas D, Seli E, Wells D (2013). Single nucleotide polymorphisms and next generation sequencing. Human gametes and preimplantation embryos: assessment and diagnosis.

[42] Treff NR, Fedick A, Tao X, Devkota B, Taylor D, Scott RT (2013). Evaluation of targeted next-generation sequencing–based preimplantation genetic diagnosis of monogenic disease. Fertil Steril.

[43] Wells D, Kaur K, Grifo J, Glassner M, Taylor JC, Fragouli E, Munné S (2014). Clinical utilisation of a rapid low-pass whole genome sequencing technique for the diagnosis of aneuploidy in human embryos prior to implantation. J Med Genet.

[44] Yin X, Tan K, Vajta G, Jiang H, Tan Y, Zhang C, Chen F, Chen S, Sheng C, Zhang C, Pan X, Gong C, Li X, Lin C, Gao Y, Liang Y, Yi X, Mu F, Zhao L, Peng H, Xiong B, Zhang S, Cheng D, Lu G, Zhang X, Lin G, Wang W (2013). Massively parallel sequencing for chromosomal abnormality testing in trophectoderm cells of human blastocysts. Biol Reprod.

[45] Fiorentino F, Biricik A, Bono S, Spizzichino L, Cotroneo E, Cottone G, Kokocinski F, Michel CE (2014). Development and validation of a next-generation sequencing–based protocol for 24 chromosome aneuploidy screening of embryos. Fertil Steril.

[46] Fiorentino F, Bono S, Biricik A, Nuccitelli A, Cotroneo E, Cottone G, Kokocinski F, Michel CE, Minasi MG, Greco E (2014). Application of next-generation sequencing technology for comprehensive aneuploidy screening of blastocysts in clinical preimplantation genetic screening cycles. Hum Reprod.

[47] Harper J, Geraedts J, Borry P, Cornel MC, Dondorp WJ, Gianaroli L, Harton G, Milachich T, Kääriäinen H, Liebaers I, Morris M, Sequeiros J, Sermon K, Shenfield F, Skirton H, Soini S, Spits C, Veiga A, Vermeesch JR, Viville S, de Wert G, Macek M (2014). Current issues in medically assisted reproduction and genetics in Europe: research, clinical practice, ethics, legal issues and policy. Hum Reprod.

[48] Yang Z, Zhang J, Salem SA, Liu X, Kuang Y, Salem RD, Liu J (2014). Selection of competent blastocysts for transfer by combining time-lapse monitoring and array CGH testing for patients undergoing preimplantation genetic screening: a prospective study with sibling oocytes. BMC Med Genomics.

[49] World Health Organization (2010). WHO Laboratory Manual for the Examination and Processing of Human Semen.

[50] Meseguer M, Herrero J, Tejera A, Hilligsoe KM, Ramsing NB, Remohi J (2011). The use of morphokinetics as a predictor of embryo implantation. Hum Reprod.

[51] Meseguer M, Rubio I, Cruz M, Basile N, Marcos J, Requena A (2012). Embryo incubation and selection in a time-lapse monitoring system improves pregnancy outcome compared with a standard incubator: a retrospective cohort study. Fertil Steril.

[52] Kuang Y, Hong Q, Chen Q, Lyu Q, Ai A, Fu Y, Shoham Z (2014). Luteal-phase ovarian stimulation is feasible for producing competent oocytes in women undergoing in vitro fertilization / intracytoplasmic sperm injection treatment, with optimal pregnancy outcomes in frozen-thawed embryo transfer cycles. Fertil Steril.

